# Potential of Auriculotherapy in Pain, Edema and Anxiety Management Following Orthognathic Surgery: A Multicenter, Randomized, Placebo-Controlled and Triple-Blind Study

**DOI:** 10.1007/s12663-024-02431-x

**Published:** 2025-01-09

**Authors:** Luigi Angelo Vaira, Giovanni Salzano, Fabio Maglitto, Umberto Committeri, Jerom R. Lechien, Miguel Mayo-Yáñez, Marco Friscia, Vincenzo Abbate, Pasquale Piombino, Luigi Califano, Giacomo De Riu

**Affiliations:** 1 Maxillofacial Surgery Operative Unit, Department of Medicine, Surgery and Pharmacy, University of Sassari, Viale San Pietro 43B, Sassari, Italy; 2 Biomedical Science Department, PhD School of Biomedical Science, University of Sassari, Sassari, Italy; 3 Department of Maxillofacial Surgery, University of Naples “Federico II”, Naples, Italy; 4 Maxillo-Facial Surgery Unit, University of Bari “Aldo Moro”, Bari, Italy; 5 Department of Laryngology and Bronchoesophagology, Mons School of Medicine, UMONS. Research Institute for Health Sciences and Technology, EpiCURA Hospital, University of Mons (UMons), Mons, Belgium; 6 Department of Otolaryngology-Head Neck Surgery, Polyclinic of Poitiers, Elsan Hospital, Poitiers, France; 7 Otorhinolaryngology, Head and Neck Surgery Department, Complexo Hospitalario Universitario A Coruña (CHUAC), A Coruña, Galicia Spain

**Keywords:** Acupuncture, Auriculotherapy, Orthognathic surgery, Maxillofacial surgery, Oral surgery, Pain, Edema, Anxiety

## Abstract

**Background:**

This multicenter, triple-blind, randomized, placebo-controlled study aims to evaluate the efficacy and safety of auriculotherapy in managing pain, edema, and anxiety following orthognathic surgical procedures.

**Materials and Methods:**

The study involved patients undergoing bimaxillary orthognathic surgery at two centers. Participants were randomized into two groups: the auriculotherapy group (AG), where vaccaria seeds were applied to six auricular points, and a placebo group (PG), where patches without seeds were applied to the same points. For ten-day post-surgery, patients underwent assessments of their pain levels, swelling, and anxiety.

**Results:**

Sixty-one patients were included (31 in the AG and 30 in the PG). The differences in pain level between the two groups were not significant until the 5-day check. Subsequently, the AG showed significantly lower levels of pain up to the 10-day follow-up. During the study period, patients in the AG consumed a significantly lower number of pain-relief medications compared to the PG (AG 18 [IQR 15.5–22.5], PG 22 [IQR 17.8–24], *p* = 0.025).

The differences between the two groups regarding the severity of the edema were not significant at all time points. The two groups did not show significant differences in terms of preoperative anxiety score. Ten days after the surgical procedure, the PG exhibited significantly higher anxiety levels compared to the AG.

**Conclusions:**

Auriculotherapy has shown promise in managing post-orthognathic surgery pain and reducing patient anxiety, while also allowing reduced medication intake. For these reasons, it could serve as a cost-effective and side-effect-free therapeutic adjunct for these patients.

## Introduction

Orthognathic surgery is among the most performed procedures in maxillofacial surgery departments worldwide. Although routine, these procedures are often accompanied by a series of inflammatory postoperative sequelae such as pain and edema [1–3]. These manifestations, although usually transient, can significantly impair the early postoperative period, contributing to patient discomfort, anxiety and prolonging recovery time [4–6].

Currently, the therapeutic approaches for managing these inflammatory sequelae encompass a wide range of options including non-steroidal anti-inflammatory [7] and steroidal [8] drugs, cooling masks [9, 10], physical [11] and low-laser [12] therapy. Despite their efficacy, these treatments often come with side effects, such as gastrointestinal complications, risk of dependency, and limited patient compliance [13]. Moreover, physical therapies mainly target symptom relief rather than the underlying inflammatory process, presenting a limitation to their overall effectiveness.

Auriculotherapy is a branch of acupuncture, characterized by the targeted stimulation of specific points on the auricle. This methodology is rooted in the concept that the auricle is a microsystem reflecting the entire body, with specific auricular points corresponding to distinct anatomical locations or physiological systems [14]. A variety of techniques are employed to stimulate these points, low-level laser therapy, or electrical impulses. An additional method involves the application of small seeds or pellets adhered to the ear, allowing patients to self-administer pressure as needed [15].

The range of potential applications for auriculotherapy is broad. Currently, it is most commonly implemented in the management of pain, both acute and chronic [16, 17]. Additionally, it has demonstrated efficacy in substance withdrawal and addiction recovery [18], stress and anxiety management [19], and as an adjunct treatment in weight loss and insomnia [20].

In the domain of maxillofacial surgery, the exploration of auriculotherapy as a therapeutic modality is still largely unexplored. Thus, only a limited number of studies, primarily investigating its role in postoperative pain and edema management following third molar extractions [21, 22], and its impact on temporomandibular joint disorders [23, 24], have been published. Notably, no research has yet investigated the application of auriculotherapy within the specific context of orthognathic surgery. This study aims to address this gap, by evaluating the efficacy and safety of auriculotherapy in managing pain, edema, and anxiety following orthognathic surgical procedures.

## Materials and Methods

### Study Design and Patients

This multicentric, prospective, placebo-controlled, and triple-blind study was conducted within the maxillofacial surgery units of the University Hospital of Sassari and the University Hospital of Naples “Federico II,” from May 2021 to January 2023. The study was approved by the ethics committee of the University Hospital of Cagliari (protocol n° PG/2021/7117) and adhered to the consolidated standards of reporting trials (CONSORT) guidelines and the ethical principles of the Declaration of Helsinki.

The study population comprised adult patients with non-syndromic dentoskeletal malocclusion due to altered maxillary growth, who were consequently undergoing bimaxillary orthognathic surgery. Exclusion criteria for the enrollment included previous head and neck surgery or radiotherapy, presence of preexisting acute or chronic pain in any anatomical region, ongoing analgesic or corticosteroid therapy prior to surgery, temporomandibular disorders, systemic, psychiatric or neurological comorbidities, alterations of the auricle, allergy or contraindications to the use of non-steroidal anti-inflammatory therapy, cortisone or antibiotics. During the study, patients were excluded in the event of bad fractures, iatrogenic section of the inferior alveolar, mental or infraorbital nerves, major bleedings during or after surgery, postoperative infection, immediate postoperative need for secondary surgery, development of allergic reactions to therapy or other factors necessitating its suspension, and other major intraoperative or postoperative complications that could influence the study’s outcomes.

Patients meeting the inclusion and exclusion criteria were scheduled for surgery. Prior to the procedure, each patient was randomly assigned to one of the two study groups using an alphanumeric spreadsheet with a 1:1 allocation ratio: the auriculotherapy group (AG) and the placebo group (PG).

### Surgical Procedure

All patients underwent virtual surgical planning tailored to the nature of the malocclusion and desired aesthetic facial outcomes [25–27]. All surgical procedures were performed under general anesthesia. In each of the two participating centers, the operations were carried out by the same experienced surgeon with more than 15 years of expertise in orthognathic surgery. Bilateral sagittal split ramus osteotomy (BSSRO), using the modified Epker’s technique, was executed through bilateral vestibular incisions from the retromolar trigone to the first molar. Le Fort 1 maxillary osteotomy was performed through a circumvestibular incision. Osteotomies were done using piezoelectric instruments (DePuy Synthes, Raynham, MA, USA). The maxilla and the mandible were repositioned under the guidance of occlusal splints made based on virtual programming. Rigid fixation was achieved using 2.0 titanium plates and screws (Stryker, Kalamazoo, MI, USA). When necessary, genioplasty was performed through an anterior mandibular vestibular incision. The surgical wounds were primarily closed, and sutures were removed 15 days after surgery.

### Pharmacological Therapy

All patients underwent antibiotic prophylaxis with amoxicillin and clavulanic acid, 1 g twice daily, from the day before the operation to 7-day post-operation. Dexamethasone 16 mg was administered 1 h before the procedure and 8 mg every 8 h after the procedure for three total doses in all cases. Patients were administered pain-relief therapy through an elastomeric pump for the first 24-h post-operation, which included ondansetron 8 mg/4 mL, ketorolac tromethamine 60 mg/2 mL, and tramadol 200 mg/2 mL. In instances where pain was still present, patients were given paracetamol 1000 mg, which could be administered up to every 6 h if required. Hilotherapy face mask was applied to all patients for the first 24 h after the procedure.

### Intervention

**Fig. 1:**
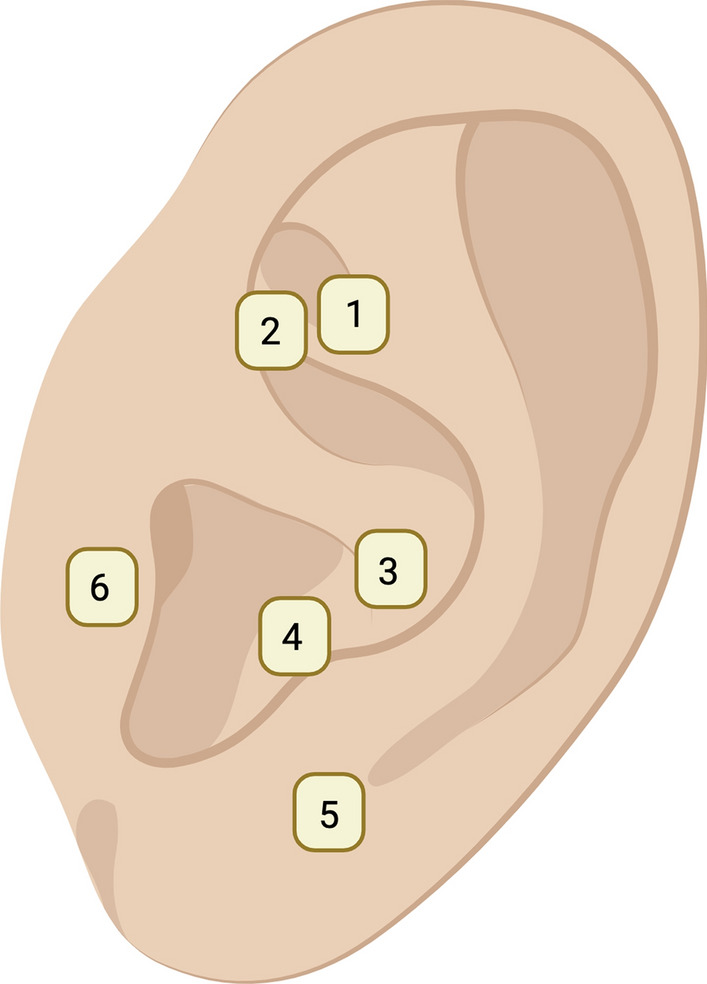
Auricular acupuncture points: (1) Shen Men, (2) Sympathetic (SNV), (3) Stomach, (4) Toothache 3, (5) Jaw and (6) Adrenal

In patients assigned to the AG, a researcher other than the operating surgeon applied vaccaria (Vaccaria hispanica) seeds at the end of the operation. These seeds were held in place by patches (Dragon Acupuncture, EasyTech Trading Pte. Ltd., Singapore). The seeds were applied to both auricles at the following points [28, 29] (Fig. 1):

1. Shen Men: This point is known to alleviate pain, anxiety, and inflammatory conditions.
2. Sympathetic: Stimulating this point can help reduce autonomic imbalances by activating the sympathetic or parasympathetic nervous system.
3. Stomach: This point is known to help alleviate dental pain.
4. Toothache 3: Applying pressure to this point can reduce pain in the lower teeth.
5. Jaw: This point helps alleviate lower tooth pain, tension, and anxiety.
6. Adrenal (suprarenal): This point is known to stimulate the adrenal glands to produce hormones, thereby helping to reduce stress.

The patients were instructed to apply pressure to each of the six points for ten seconds four times daily. They were further advised to stimulate these points at the onset of pain. If symptom relief was observed following the maneuver, they could repeat it as necessary. In the absence of any relief, patients were encouraged to resort to as-needed pain-relief therapy. The seeds remained in place until suture removal, which took place ten-day post-surgery.

For the patients in the PG, identical patches were positioned at the same six points as those used for the AG patients at the conclusion of surgery, although these did not contain the vaccaria seeds. Furthermore, these patients were given the same instructions for point stimulation and followed identical procedures to those implemented in the AG.

### Outcomes

Each participant was evaluated on three clinical factors: pain, swelling, and anxiety levels. In each clinical center, a single researcher, unaware of the patients’ group assignments, performed all these measurements.

To evaluate pain, patients were given a form and asked to quantify their pain using a visual analog scale (VAS), ranging from 0 (representing no pain) to 10 (indicating intolerable pain). This assessment was conducted at 6-, 12-, and 24-h following surgery, and then at 24-h intervals until 10-day post-surgery. Additionally, patients were asked to document the consumption of any pain-relief medication during the observation period.

For swelling quantification, the method described by Ustun et al. [30] was utilized. Three connecting lines were considered as follows: Line 1: from the outer corner of the eye to the mandibular angle; Line 2: from the tragus to the corner of the mouth; Line 3: from the tragus to the midpoint of the chin. Measurements were taken before surgery to establish a baseline, and then at 1, 3, and finally at 10-day post-surgery. The sum of the six preoperative measurements (three for each side) served as the norm. The extent of the swelling was calculated by subtracting the total preoperative measurements from the total of six postoperative measurements at each evaluation point. The evaluation was performed before surgery to establish a baseline and subsequently at one, three- and ten-day post-surgery.

Levels of anxiety were assessed using the short version of the Spielberger State-Trait Anxiety Inventory (STAI) [31]. The STAI is a validated and widely used questionnaire designed to gauge anxiety levels through 10 forced-choice items. The scores from this questionnaire can range from 10, indicating normal levels of anxiety, to 40, suggesting high levels of anxiety. For the purposes of our analysis, the difference between the preoperative scores and those obtained 10-day post-operation were considered.

### Statistical Analysis

The determination of the sample size was carried out utilizing G*power 3.1 software (Heinrich Heine University Dusseldorf, Dusseldorf, Germany). The parameters used for this calculation were drawn from the study conducted by Sampaio et al. [32] and Vaira et al. [22] for third molar surgery: with a power of 80%, a margin of error of 5%, and Cohen’s D at 0.51. Consequently, a minimum of 52 patients (26 for each group) were needed for the study.

Statistical analysis was accomplished using the freely available and open-source software Jamovi, version 2.3.18.0, accessible online at www.jamovi.org. Data are presented either as absolute numbers or as a percentage of the total. Descriptive statistics for quantitative variables are expressed as means ± standard deviation or median [interquartile range—IQR].

The one-way ANOVA and the Fisher’s exact test were used to assess differences between the two groups in terms of continuous or categorical variables, respectively. The threshold for statistical significance was established at *p* < 0.05, with a confidence interval of 95%.

## Results

**Fig. 2:**
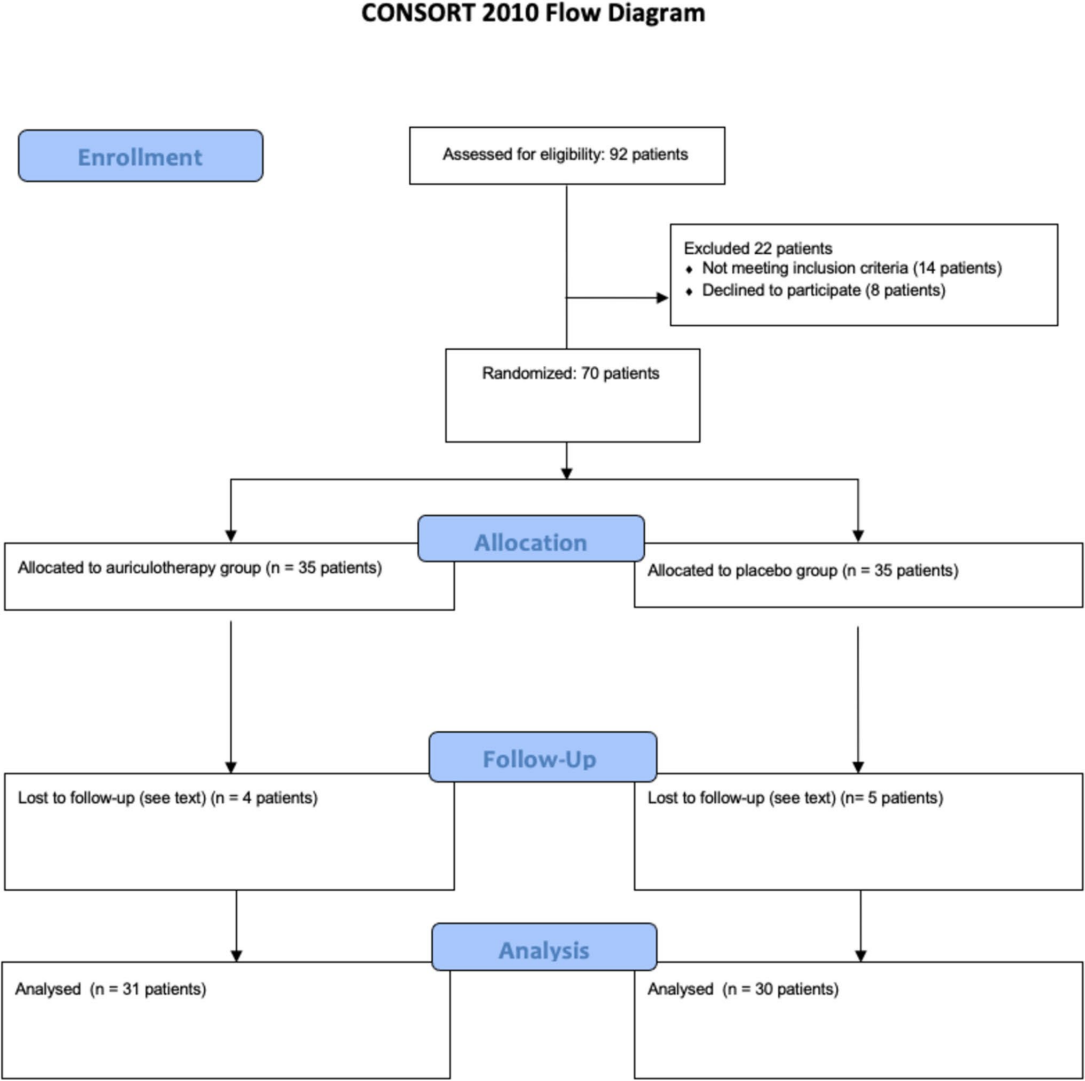
CONSORT flowchart

The study enrolled a total of 70 patients who met the inclusion criteria and were subsequently divided into two study groups, each containing 35 patients. Following the procedure and during the follow-up period, nine patients were excluded for the following reasons: bad fracture (one case), postoperative infection (two cases), osteotomy revision (one case), allergic reaction to therapy (one case), and incomplete data or inconsistent follow-up (four cases). Consequently, the analysis was conducted on data from 31 patients in the AG and 30 in the PG (Fig. 2). Table 1 presents a summarized overview of the characteristics of the included patients. No significant differences were noted with respect to gender, age, type of malocclusion and surgical procedure between the two groups (Table 1). No side effects related to auriculotherapy were detected in the patients included in the study.

**Table 1:** Patients’ characteristics

	Auriculotherapy group (N: 31)	Placebo group (N: 30)	*p*-value
Gender			
Male	15 (48.4%)	16 (53.3%)	0.8*
Female	16 (51.6%)	14 (46.7%)	
Age (median IQR)	31 [25.5–35]	28.5 [26–32.5]	0.480**
Type of malocclusion			
Class II	12 (38.7%)	12 (40%)	0.895*
Class III	19 (61.3%)	17 (56.7%)	
Asymmetry	0 (0%)	1 (3.3%)	
Type of surgery			
Bimaxillary	12 (38.7%)	15 (50%)	0.444*
Bimaxillary + mentoplasty	19 (61.3%)	15 (50%)	

^*^Fisher’s exact test, **One-way ANOVA

**Fig. 3:**
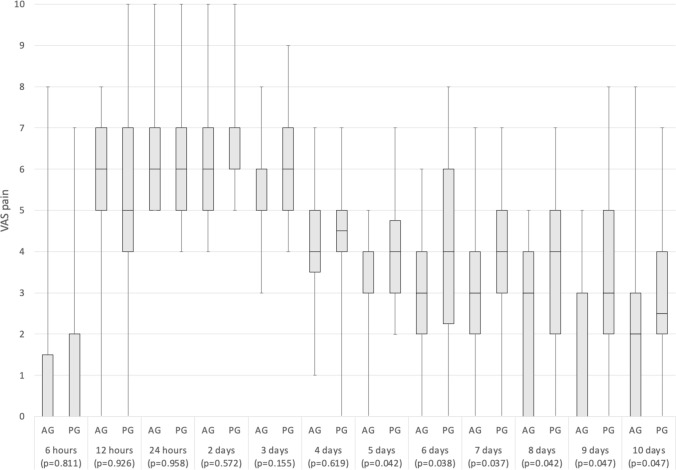
Comparison of the pain VAS scores between the two study groups during the observation period

At baseline, none of the patients reported pain. After the surgery, for both groups, the time points at which pain was most elevated were at 24 (AG VAS 6 [IQR 5–7], PG VAS 6 [IQR 5–7]; *p* = 0.958) and 48 h (AG VAS 6 [IQR 5–7], PG VAS 6 [IQR 6–7]; *p* = 0.572). The differences between the two groups were not significant until the 5-day check. Subsequently, the AG showed significantly lower levels of pain up to the 10-day follow-up [Fig. 3] [Table 2]. During the study period, patients in the AG consumed a significantly lower number of pain-relief medications compared to the PG (AG 18 [IQR 15.5–22.5], PG 22 [IQR 17.8–24], *p* = 0.025).

**Table 2:** Clinical analysis results

	Auriculotherapy group (N: 31)	Placebo group (N: 30)	*p*-value
Pain (VAS) median [IQR]			
Preoperative	0 [0–0]	0 [0–0]	1
6 h	0 [0–1.5]	0 [0–2]	0.811
12 h	6 [5–7]	5 [4–7]	0.926
24 h	6 [5–7]	6 [5–7]	0.958
2 days	6 [5–7]	6 [6, 7]	0.572
3 days	5 [5, 6]	6 [5–7]	0.155
4 days	4 [3.5–5]	4.5 [4, 5]	0.619
5 days	4 [3, 4]	4 [3–4.75]	0.042
6 days	3 [2–4]	4 [2.25–6]	0.038
7 days	3 [2–4]	4 [3–5]	0.037
8 days	3 [0–4]	4 [2–5]	0.042
9 days	3 [0–4]	3 [2–5]	0.047
10 days	2 [0–3]	2.5 [2–4]	0.047
Pain-killer medications (N°) Median [IQR]	18 [15.5–22.5]	22 [17.8–24]	0.025
Edema (mm) median [IQR]	31 [25.5–35]	28.5 [26–32.5]	0.480
24 h	709 [643–755]	667 [622–721]	
Difference with preoperative	98 [85.5–111]	89.5 [85–99.5]	0.179
3 days	743 [690–795]	692 [658–749]	
Difference with preoperative	135 [116–146]	125 [113–133]	0.103
10 days	623 [558–661]	576 [545–642]	
Difference with preoperative	13 [10–15]	16 [12–22]	0.656
Anxiety (STAI score) median [IQR]			
Preoperative	17 [14–20.5]	17.5 [15–19]	0.803
10 days	25 [24–27.5]	28 [25.3–30.8]	
Difference with preoperative	9 [6.5–10]	11.5 [8.25–13]	0.033

The differences between the two groups regarding the severity of the edema were not significant at all time points (Table 2).

The two groups did not show significant differences in terms of preoperative STAI score. Ten days after the surgical procedure, the PG exhibited significantly higher anxiety levels compared to the AG (Table 2).

## Discussion

Acupuncture has shown promising efficacy in the management of pain and edema following fractures in the past [33, 34]. However, its routine application is challenging since it necessitates direct involvement by healthcare professionals, thereby increasing the number of clinical visits for the patient. In this context, auriculotherapy can be a more viable alternative due to its simpler execution, enabling patient self-management. Nonetheless, the application of auriculotherapy in orthognathic or traumatological surgery remains a largely uncharted area. The only study published to date in the traumatology domain found a significant reduction in pain, heart rate, and anxiety in elderly patients with hip fractures undergoing auriculotherapy [35]. On the other hand, the scant research on auriculotherapy’s effects for pain management after outpatient oral surgery has yielded inconsistent results, and there is no consensus on the regimen and timing of administration [22, 32].

In this study, patients who underwent auriculotherapy reported significantly lower pain levels starting from the fifth postoperative day until the end of the observation period. Furthermore, they required a significantly fewer number of pain-relief medications compared to the PG.

Scientific exploration into the mechanisms underpinning auriculotherapy pain-relief effect is ongoing [36, 37]. Current theories suggest the stimulation of auricular points influences the activity of both the trigeminal and vagus nerves. This activity is thought to be conveyed to shared brainstem nuclei, resulting in diverse systemic effects. Notably, anti-inflammatory and pain-relief effects are believed to be mediated via efferent systems of the vagus nerve, demonstrating a potential mechanism by which auriculotherapy may modulate inflammatory processes [37]. Another potential mechanism is related to auriculotherapy’s ability to induce the release of endorphins, thereby increasing the pain threshold [38]. Compared to the stimulation of auricular points with low-laser therapy, which has not shown significant effects on pain control after third molar extraction [32], the use of Vaccaria seeds offers the advantage of allowing the patient to manage it directly. They can apply the stimulation precisely when pain arises, rather than only at predetermined times. The effect of auriculotherapy in this study was evident only from the fifth postoperative day. The initial postoperative days are typically characterized by intense inflammation, which might overshadow the subtle effects of auriculotherapy. As the inflammation diminishes, the analgesic effects of the auriculotherapy become more pronounced and thus, more clinically observable.

In addition to the mechanisms previously mentioned, the anti-edematous effect of auriculotherapy might be mediated by the potential capacity of stimulating the “adrenal” auricular point to induce cortisol production [29]. However, this hypothesis has never been substantiated by studies monitoring blood cortisol levels after auriculotherapy. In the present study, auriculotherapy proved ineffective in controlling postoperative edema, and these findings align with those reported by other authors regarding oral surgery procedures [22, 32, 39]. Unlike edema, pain is not solely mediated by inflammatory processes, but also has a significant central component on which auriculotherapy can act [40]. This is why different efficacies might be achieved in managing these two symptoms.

Controlling anxiety in orthognathic surgery patients is essential for better outcomes. Preoperative education and clear communication significantly reduce anxiety levels, as patients with better knowledge report lower anxiety [41]. Psychosocial techniques like cognitive-behavioral therapy and relaxation training also help manage anxiety and improve recovery [42]. Administering anxiolytics preoperatively can decrease both anxiety and the need for postoperative analgesics [43]. Systematic relaxation techniques are effective in reducing anxiety and pain [44]. Educational workshops enhance patient readiness and reduce anxiety by providing comprehensive understanding and practical resources [45]. Lavender oil inhalation before surgery did not show significant anxiolytic effects [46].

The mechanism by which auriculotherapy reduces anxiety has been investigated in animal models. It is believed to be associated with an increase in protein synthesis essential for synaptic plasticity in the hippocampus [47]. Its efficacy in addressing anxiety has been demonstrated in oral surgery [21, 48] and numerous other conditions [49–51]. Notably, anxiety is a frequent aftermath of orthognathic surgeries, bearing significant aesthetic and functional implications [52]. In the current study, patients who underwent auriculotherapy displayed notably lower anxiety levels 10-day post-procedure compared to those in the PG. Recognizing and addressing this reduction in anxiety is crucial, as alleviating such emotional distress can greatly enhance postoperative recovery and overall patient well-being.

This study boasts significant strengths: the randomized design, the multicentric setting, and the triple-blind approach ensure the reliability of the results, which are further supported by the power statistical analysis. To minimize the risk of bias introduced by having two different surgical teams operate on the patients, enrolled individuals were randomized into two groups, ensuring an even distribution of patients from both centers in each group. Differences in clinical parameters evaluated between the two centers were not statistically significant. Another potential limitation is the challenge in maintaining patient blinding, as those in the PG might perceive the absence of the seed beneath the patch. However, this is the only way to ensure that no stimulus is applied to the auricle. Seedless patches are still considered a reliable sham control method and are widely used in control groups [21, 22, 53].

## Conclusions

This research underscores the potential of auriculotherapy as a supplementary approach to managing pain following orthognathic surgery, while pharmacological therapy remains paramount for pain control in the initial postoperative days as in this stages auriculotherapy failed to achieve significant effects in pain control. On the contrary, in the later stages of recovery, auriculotherapy has proven effective in pain management, allowing patients to reduce their medication intake. No effect were noted in the control of edema. Notably, patients’ anxiety levels also witnessed a substantial reduction. On the downside, auriculotherapy did not show a notable influence on postoperative edema management. An advantage of this technique, leveraging vaccaria seeds for auricular point stimulation, lies in its patient-centric application, obviating the need for frequent medical follow-ups. This patient autonomy can bolster adherence and potentially make auriculotherapy a cost-efficient adjunct in the therapeutic regimen for those undergoing orthognathic procedures.

## Data Availability

The data that support the findings of this study are available on request from the corresponding author [LAV].
